# Reactive oxygen species regulation of ferroptosis, autophagy, apoptosis, and immunogenic cell death in cancer

**DOI:** 10.3389/fphys.2026.1908766

**Published:** 2026-08-10

**Authors:** Seema Kumari, Sudhir Kumar Rai

**Affiliations:** 1 Department of Biotechnology, Dr. B. R. Ambedkar University, Srikakulam, Andhra Pradesh, India; 2 Laboratory for Cancer Variant Biology, Department of Quantitative Health Sciences, John A. Burns School of Medicine, University of Hawaii at Manoa, Honolulu, HI, United States

**Keywords:** apoptosis, autophagy, cancer therapy, ferroptosis, ICD, ROS

## Abstract

Reactive oxygen species (ROS) are produced during metabolism through mitochondrial oxidative phosphorylation and NADPH oxidase activity. In cancer, ROS exhibit a paradoxical, concentration-dependent dual role. At low to moderate levels, ROS promote tumor cell survival, EMT, and metastasis, and at high levels, ROS overwhelm antioxidant defenses and induce regulated cell death (RCD); ferroptosis, autophagy, apoptosis, and immunogenic cell death (ICD) have emerged as significant ROS-dependent targets in cancer therapy. This review summarizes recent advances in ROS-controlled ferroptosis, autophagy, apoptosis, and ICD, emphasizing the roles of the GPX4/SLC7A11, AMPK/mTORC1/Beclin-1, Bcl-2/Bax/cytochrome c, and damage-associated molecular pattern (DAMP)-mediated signal cascade, their crosstalk, and therapeutic potential for targeting tumor redox vulnerabilities.

## Highlights

- Discuss ROS-driven ferroptosis, autophagy, apoptosis, and ICD in cancer
- Highlights GPX4/SLC7A11, AMPK/mTOR, Bcl-2/Bax, and DAMPs as ROS-regulated pathways
- Targeting ROS-mediated cell death can overcome therapy resistance

## Introduction

**Figure 1:**
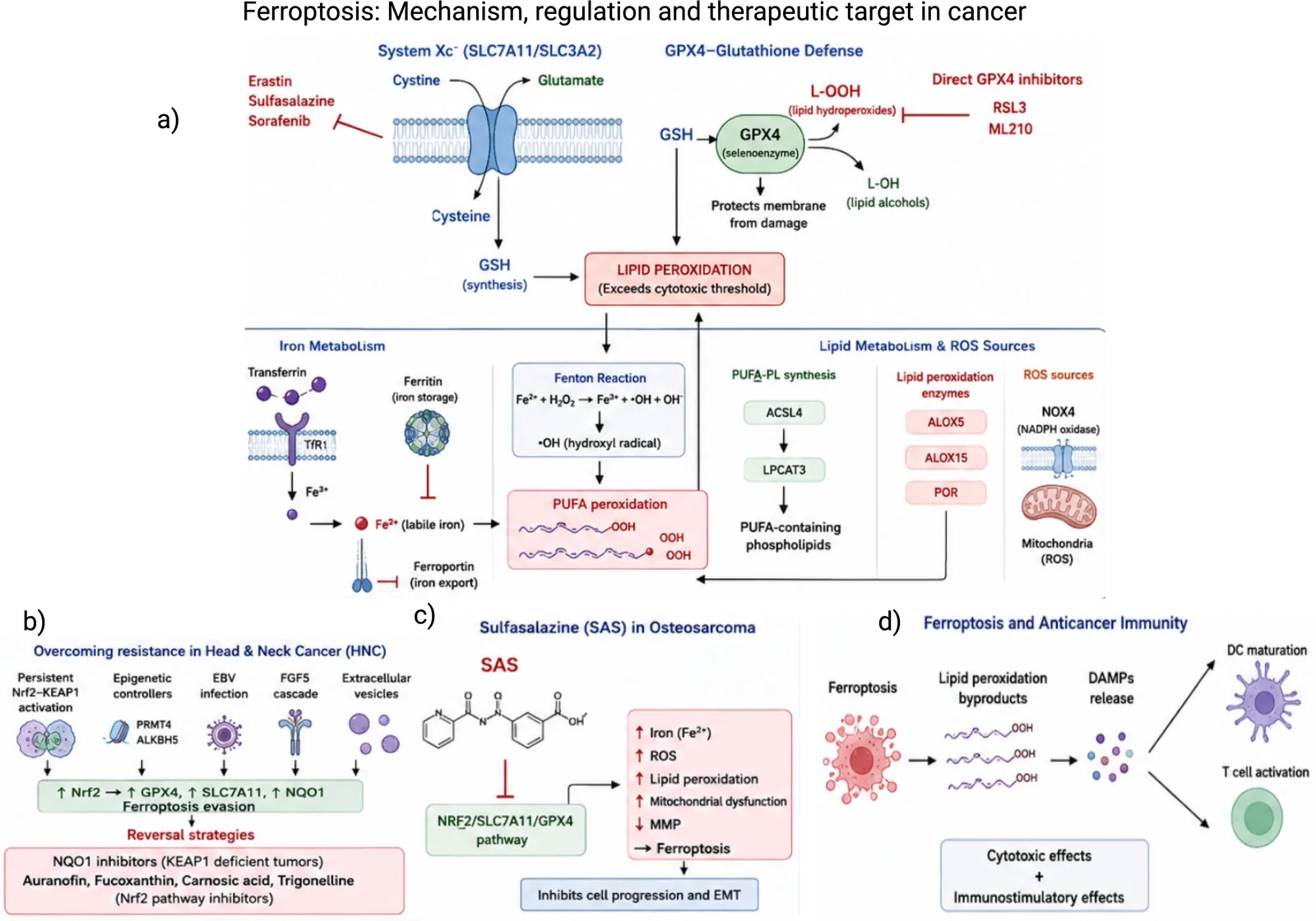
The mechanism, regulation, and therapeutic advancements of ferroptosis in cancer. It is driven by iron-mediated lipid peroxidation and regulated by Xc^−^–GSH–GPX4 and FSP1 antioxidant pathways. Critical regulators include NRF2, p53, p38 MAPK, ACSL4, LPCAT3, ALOX5/15, POR, and iron metabolism. Advancements in therapeutic strategies targeting ferroptosis have improved the tumor cytotoxicity, overcome therapy resistance, and stimulate antitumor immunity.

Reactive oxygen species (ROS) are an array of derivatives liberated during metabolic processes including hydrogen peroxide (H_2_O_2_), superoxide anion (O_2_^.−^), hydroxyl radicals (OH^.^), singlet oxygen (¹O_2_), and nitric oxide (NO^.^). ROS molecules control cellular redox signal and act as secondary messengers in governing cell proliferation, differentiation, and survival. ROS are generated during electron transport chain (ETC) at complexes I and III and through enzymatic sources of NADPH oxidases (NOXs) and xanthine oxidase (XO) and leaked through electrons from mitochondria (de Almeida et al., 2022). In addition, NADPH oxidases (NOX1–5 and DUOX1 and 2) are also major sources of ROS with NOX4 often overexpressed in tumors and chronic elevated H_2_O_2_ that encourages tumor cell survival and oncogenic signal (Konaté et al., 2020). The preservation of ROS homeostasis is regulated through a complex network of antioxidant defense mechanism controlled by a series of enzymes superoxide dismutase (SOD), catalase, glutathione peroxidases (GPXs), and thioredoxins alongside the non-enzymatic scavenger glutathione (GSH) (Chen et al., 2025) (**Figure 1a**). During cancer development, dysregulated ROS levels disrupt redox homeostasis and enhance metastasis by DNA lesions, genetic instability, metabolic transformation, and activation of mitogenic (RAS-RAF-MEK-ERK and PI3K-Akt) signal pathways. Mitochondria dysfunction further promotes tumorigenesis via kinase stimulation, HIF-1α stabilization, neovascularization, and EMT (Akter et al., 2026), while enhanced antioxidant defense systems by nuclear factor erythroid 2-related factor 2 (NRF2), cystine-glutamate antiporter system Xc^−^ (SLC7A11-SLC3A2) (Jyotsana et al., 2022), and thioredoxin avert critical ROS threshold from cytotoxic levels. However, persistent ROS signals activate stress-associated pathways by engaging apoptosis signal-regulating kinase 1 (ASK1), along with PARP- and ATG4-mediated autophagy. Consequently, ROS play a dual role in regulating tumor cell survival and death (Zhao et al., 2023). In cancer, oxidative stress is alleviated via NRF2-KEAP1 signaling, by oxidation of KEAP1 and stabilization of NRF2, inducing cytoprotective genes such as SLC7A11/NQO1/HO-1/GCLC and GPX2. Persistent KEAP1/NRF2 mutations in lung adenocarcinoma were reported to regulate constitutive antioxidant signaling and cause chemoresistance using thioredoxin/thioredoxin reductase, peroxiredoxins, and the pentose phosphate pathway (PPP), by delivering NADPH for GSH regeneration (Chen et al., 2026). Current studies in the role of ROS in cancer distinguishes ROS as “ROS threshold”, permitting selective antitumor effect by overwhelming antioxidant defenses through pro-oxidant therapies, ROS-producing nanoparticles, and targeted inhibition of antioxidant systems with limited impact on healthy cells possessing robust redox homeostasis (Agrawal et al., 2023). Sublethal concentrations of ROS promote tumor growth by activating mitogenic signals, NF-κB, and HIF-1α, though a high level of ROS induces oxidative damage and triggers regulated cell death (Aggarwal et al., 2019). Among the ROS-regulated cell death mechanisms, there are four significant pathways: (1) ferroptosis, an iron-dependent lipid peroxidation (Liu et al., 2020); (2) autophagy, a lysosomal degradation pathway with a microenvironment dependent pro-survival or pro-death role (Lee et al., 2023); (3) caspase-dependent apoptosis (Wu et al., 2022); and (4) damage-associated molecular patterns (DAMPs) triggered immunogenic cell death (ICD) (Dou et al., 2024). This review addresses how ROS regulate cell death mechanism, their molecular interconnections, and their therapeutic implications in tumor development.

## ROS regulation of ferroptosis

Ferroptosis is an iron-dependent, non-apoptotic cell death caused by peroxidation of PUFAs, including arachidonic acid and adrenic acid esterification in membrane phospholipids due to dysregulated redox balance. The GPX4-GSH and FSP1 antioxidant defense are major regulators that protect cancer cells from ferroptotic damage (Chen et al., 2024). Targeting these defenses promotes lipid peroxidation beyond a cytotoxic threshold, thus eliminating therapy-resistant tumor cells. Ferroptosis has emerged as a significant therapeutic strategy for overcoming resistance to therapy and enhancing effectiveness of immunotherapies.

Glutathione peroxidase 4 (GPX4), a selenoenzyme, controls ferroptosis by reducing lipid hydroperoxides (L-OOH) to lipid alcohols (L-OH) and averting membrane damage. The GPX4 activity depends on GSH, synthesized from cysteine imported through the Xc^−^ (SLC7A11/SLC3A2) transporter (Weaver and Skouta, 2022). Erastin, sulfasalazine, and sorafenib inhibitors of Xc^−^ deplete cysteine and GSH, leading to GPX4 inactivation and accumulation of lipid peroxide, and trigger ferroptosis. Direct GPX4 inhibitors such as RSL3 and ML210, by directly targeting the GPX4 active site, independent of GSH depletion, cause ferroptosis (Li et al., 2022). Labile redox-active Fe^2+^ is required for ferroptosis, catalyzing the Fenton reaction to initiate PUFA peroxidation via hydroxyl radicals. Additionally, enhanced iron uptake and impaired iron storage, regulated by transferrin receptor 1 (TfR1), ferritin, and ferroportin, increase lipid peroxidation and trigger ferroptosis. ACSL4/LPCAT3 generate PUFA-containing phospholipids, whereas ALOX5, ALOX15, and POR catalyze lipid peroxidation during ferroptosis. NOX4- and mitochondria-derived ROS also add up to lipid peroxidation cascades (Hemmati et al., 2025).

### Regulatory signaling pathways

Ferroptosis is synchronized by a complex interplay of oncogenic and tumor-suppressive signaling pathways, among which NRF2-SLC7A11-GPX4 is the dominant anti-ferroptotic transcriptional setting, whereas p53, a tumor, exhibits a dual role as it can inhibit ferroptosis by inducing CDKN1A (p21), but more often, it promotes ferroptosis by transcriptional suppression of SLC7A11 and activation of SAT1 and GLS2. p38 stimulation by ROS controls the process of ferroptosis through DPP4 (Dipeptidyl Peptidase 4) and EMP1 (Epithelial Membrane Protein 1) (**Figure 1a**). The mevalonate pathway regulates GPX4 function and ferroptosis susceptibility (Lee et al., 2025).

### Advancements in cell death pathway-based therapies

Ferroptosis signifies a potential strategy against resistance to therapy as reported in head and neck cancer (HNC). Persistent activation of Nrf2-KEAP1 enhances ferroptosis evasion through stimulation of GPX4, SLC7A11, and NQO1 while epigenetic controllers including PRMT4, ALKBH5, EBV infection, FGF5 cascade, and extracellular vesicles also enhance Nrf2-mediated resistance. Approaches including NQO1-based targeted therapy in KEAP1-deficient tumors and utilization of auranofin, fucoxanthin, carnosic acid, and trigonelline, an inhibitor of Nrf2, signal reverse resistance to ferroptosis (Lee et al., 2025). Furthermore, utilizing network pharmacology, MD, and mitochondrial membrane potential (MMP) analysis demonstrated that sulfasalazine (SAS) inhibits osteosarcoma cell progression and EMT by inducing ferroptosis through suppression of NRF2/SLC7A11/GPX4 signaling where there is accumulation of iron, ROS stress, lipid peroxidation, and mitochondrial dysfunction, making SAS a novel therapeutic approach in osteosarcoma (Qin et al., 2025) (**Figures 1b, c**). In addition, ferroptosis stimulates anticancer immunity via DAMP release, while mitochondria-targeted ferroptosis inducers augment lipid peroxidation, increasing therapeutic efficacy and selectivity. Ferroptosis intersects with ICD, via lipid peroxidation by-products functioning as DAMPs, and exerts cytotoxic and immunostimulatory effects, while mitochondria-targeted ferroptosis inducers stimulate lipid peroxidation in mitochondrial membranes, enhancing effectiveness and reducing off-target effects (**Figure 1d**).

## ROS regulation of autophagy

Autophagy is defined as a conserved lysosome-mediated cellular recycling process that degrades damaged organelles, misfolded proteins, and cell debris through macroautophagy, microautophagy, and chaperone-mediated autophagy. Autophagy is begun by stress-induced inhibition of mTORC1 and activation of the ULK1 complex, which stimulates the PIK3C3/BECN1/ATG14/AMBRA1/VPS15/NRBF2 complex to make PtdIns3P and nucleate phagophores. Consequent ATG7- and ATG3-mediated lipidation of LC3 drives autophagosome formation, cargo sequestration, and lysosomal degradation through processes such as mitophagy, ferritinophagy, pexophagy, and xenophagy (Khandia et al., 2019). Autophagy eliminates mitochondria and peroxisomes’ ROS-generating subcellular components. ROS-induced autophagy is regulated through ER mitochondria contact sites (MAMs), the thioredoxin (TXN/TXNRD1) redox system, NADPH metabolism, and redox-sensitive proteins PRDX1/2/6 and GSTP1 (Zhou et al., 2022).

### ROS as autophagy regulators via AMPK/mTORC and mitophagy

ROS regulate autophagy at gene expression and post-translational levels. In gene expression regulation, ROS activate NRF2, which controls the expression of autophagy receptor of p62/SQSTM1; the p62-KEAP1-NRF2 feedback loop is a significant node connecting redox stress to autophagy gene expression. ROS activate AMPK, inhibit mTORC1, and stimulate ULK1, thereby initiating autophagosome formation (Xiao et al., 2025). The PI3KC3 complex consists of VPS34, Beclin-1/ATG6, and ATG14, which are recruited by ULK1 to PI3P, and facilitates ATG recruitment and autophagosome expansion. ROS regulate autophagy through ATG4B inhibition and Beclin-1 activation, while PTEN oxidation counteracts autophagy via PI3K/Akt/mTORC1 signaling (Hasan et al., 2022). ROS also regulate autophagy via the PTEN-PI3K/Akt-mTOR, ATM-TSC2-mTOR, AMPK, and Beclin-1 pathways (Gouda et al., 2025). ROS induce autophagy by suppressing mTOR, releasing Beclin-1 from Bcl-2, and activating the PIK3C3 complex through redox-sensitive regulators such as ATM, AMPK, JNK, and TRAF6.

Mitophagy is the elimination of dysfunctional (ROS damaged) mitochondria to maintain mitochondrial quality, metabolic homeostasis, and redox balance. Regulated primarily by the PINK1-Parkin and BNIP3/NIX pathways, mitophagy limits ROS accumulation, ferroptosis, inflammation, senescence, and tumor progression, whereas defective mitophagy contributes to mitochondrial dysfunction and neoplasia (Macleod, 2020). A study reported that supplementation with urolithin A (UA), enhances mitophagy-mediated immune rejuvenation by expanding naïve-like CD8^+^ T cells, refining mitochondrial function and fatty acid oxidation, and promoting anti-inflammatory and immune-enhancing responses, thereby helping counteract immunosenescence and inflammaging (Denk et al., 2025).

### Duality of autophagy in cancer

Autophagy in early tumorigenesis suppresses tumor initiation by eradicating damaged organelles and prevents genetic ablation. In breast and ovarian cancer, Beclin-1 loss is associated with tumorigenicity consistent with an autophagy-suppression characteristic. In later stages, autophagy maintains tumor survival under metabolic stress, hypoxia, and chemoradiotherapy; thus, autophagy-driven therapy resistance has prompted the use of chloroquine and hydroxychloroquine as adjuvants to enhance anticancer therapy efficacy. The threshold ROS levels affect autophagy, because at low to moderate ROS levels, protective autophagy is induced, eliminating damaged components, and helps in cell survival; at persistently high ROS levels, excessive or dysregulated autophagy contributes to autophagic cell death (type II programmed cell death). Recent data show that autophagy show duality in lung cancer, functioning as either a tumor-promoting survival mechanism or a cell death pathway. Therapeutic modulation of autophagy using inhibitors (3-MA) or inducers (baicalin, CHEPS, and PPVI) suppresses tumor growth, overcomes drug resistance, and enhances apoptosis through ROS, AMPK, and PI3K/AKT/mTOR-mediated signaling (Xue et al., 2026). A recent study suggested that Guizhi Fuling capsule GZFL alleviated peripheral nerve injury by reducing IL-6-mediated inflammation, enhancing autophagy, downregulating ROS stress, and improving cell function through modulation of the IL-6/AKT/mTOR signaling pathway (Fu et al., 2025). ROS-regulated autophagy and mitophagy maintain cellular and mitochondrial homeostasis, influence cancer cell survival or death, and represent promising therapeutic targets for overcoming tumor progression, therapy resistance, and age-related dysfunction.

## ROS regulation of immunogenic cell death

ICD eradicates tumor cells and activates adaptive antitumor response by releasing tumor antigen and creating an immunogenic memory, thus permitting the combination of cytotoxic treatments with immunotherapy to transform immunologically “cold” tumors into immune-infiltrated “hot” tumors. ICD is a spatiotemporal harmonized emission of DAMPs; calreticulin, which triggers phagocytosis along with this ICD, induces ATP, HMGB1, HSPs, type I IFNs, and CXCL10 release, promoting dendritic cell recruitment, antigen presentation, and immune activation. In lung cancer, agents such as (−)-Guaiol, iridium complexes, sea hare hydrolysates, and dihydroartemisinin/cisplatin induce ICD through ROS-, ER stress-, autophagy-, and apoptosis-related pathways, enhancing the efficacy of immune checkpoint inhibitors and offering a promising strategy to overcome immunotherapy resistance (Xue et al., 2026).

### ROS and ER stress as drivers of ICD

ROS and ER stress stimulate ICD. Compounds like anthracyclines including doxorubicin, mitoxantrone, and photosensitizers cause intense mitochondrial ROS generation and ER stress, leading to ROS-driven CRT translocation from the ER lumen to the cell surface and via PERK-eIF2α phosphorylation, caspase-8 activation, and vesicular trafficking, resulting in ER-targeted ROS strongly promoting ICD (Fu et al., 2025). Photodynamic therapy (PDT) is a potent ICD inducer as type II photosensitizers generate singlet oxygen (¹O_2_) in the ER, triggering CRT exposure, ATP secretion, and HMGB1 release. Sonodynamic therapy (SDT) utilizes ultrasound-activated sonosensitizers to produce ROS, and nanoplatform designs in combination with SDT with PDT have achieved synergistic effect in solid tumors. Radiation therapy induces ICD through ROS-mediated DNA damage and type I interferon signaling via the cGAS-STING pathway (Tao et al., 2025; Khorrami et al., 2026). CRT recruits and activates immature DCs for TAA phagocytosis, while ATP and HMGB1 enhance inflammasome activation, cytokine release, and antigen presentation. Mature DCs then prime CD8^+^ and CD4^+^ T cells, cytotoxic effect, and immune memory. A study identified HMGB2 as a crucial mediator of oxaliplatin-induced calreticulin translocation and ferroptosis. Both processes are controlled by the nuclear exporter XPO1 (exportin-1) and regulated through XPO1-dependent nuclear export in multiple myeloma (Blair et al., 2024). Cationic spherical polypeptides (CSPs) and manganese oxide nanoparticles enhance ROS generation, triggering ICD through CRT exposure, HMGB1 release, and ATP secretion while promoting antitumor immune responses by exploiting TME acidity and high oxidative stress (Guo et al., 2025). Thus, ICD inducers such as anthracyclines, oxaliplatin, PDT, hypericin, bleomycin, and ROS-generating nanoparticles enhance immune priming and, in combination with anti-PD-1/PD-L1 and anti-CTLA-4 therapies, may improve the outcome.

## Crosstalk between ROS-regulated cell death signaling and therapeutic implications

Ferroptosis, autophagy, and apoptosis are interconnected and dependent through common signaling networks. NRF2 is bound by KEAP1 and targeted for proteasomal degradation; during oxidative stress, NRF2 dissociates from KEAP1, migrates to the nucleus, and activates ARE genes, providing defense against oxidative stress (Zhang et al., 2024). In ferroptosis, NRF2 suppresses lipid peroxidation by SLC7A11, GPX4, HO-1, NQO1, and ferritin, and stabilizes GSH to prevent iron-mediated lipid peroxidation conferring resistance to ferroptosis (Yan et al., 2023). In autophagy, NRF2 interacts with p62/SQSTM1, which binds KEAP1 and stabilizes NRF2, to form a positive feedback loop that enhances antioxidant defense and survival. In apoptosis, NRF2 decreases intracellular ROS levels, and suppresses apoptosis. The tumor suppressor p53 integrates signals from DNA damage, free radical stress, and oncogenic activation; p53 promotes ferroptosis by repressing SLC7A11, decreasing cystine uptake and GSH synthesis, and increasing lipid ROS accumulation. However, p53 inhibits ferroptosis through induction of CDKN1A (p21) in a context-dependent manner (Armeli et al., 2024). p53 transcriptionally activates BAX, PUMA, NOXA, and FAS, activating the intrinsic apoptotic pathway. In autophagy, p53 activates AMPK/DRAM1 and inhibits mTOR to regulate context-dependent autophagy (Wang X. et al., 2025). Activated AMPK inhibits mTORC1, inducing autophagic degradation and recycling. Autophagy promotes ferroptosis through NCOA4-associated ferritinophagy, increasing labile iron and lipid peroxidation. AMPK controls ferroptosis through the inhibition of ACC1/ACC2 and alters PUFA availability for lipid peroxidation. mTOR activation suppresses autophagy and ferritin degradation, limiting ferroptosis while promoting cell survival (Wang D. et al., 2025). JNK is activated by oxidative stress, inflammatory cytokines, and DNA damage, and persistent JNK activation phosphorylates BCL-2 and BIM, interrupts anti-apoptotic complexes, and promotes mitochondrial apoptosis. JNK induces autophagy by disrupting the Beclin-1/BCL-2 complex. In ferroptosis, lipid ROS activate JNK, amplifying oxidative stress and promoting ferroptosis and apoptosis (Yang and Yao, 2015). Elevated mtROS promote lipid peroxidation and ferroptosis while triggering intrinsic apoptosis. Mitophagy removes damaged mitochondria to limit ROS and maintain homeostasis, whereas impaired mitophagy increases ROS, enhancing both ferroptosis and apoptosis (Lin et al., 2024).

**Figure 2:**
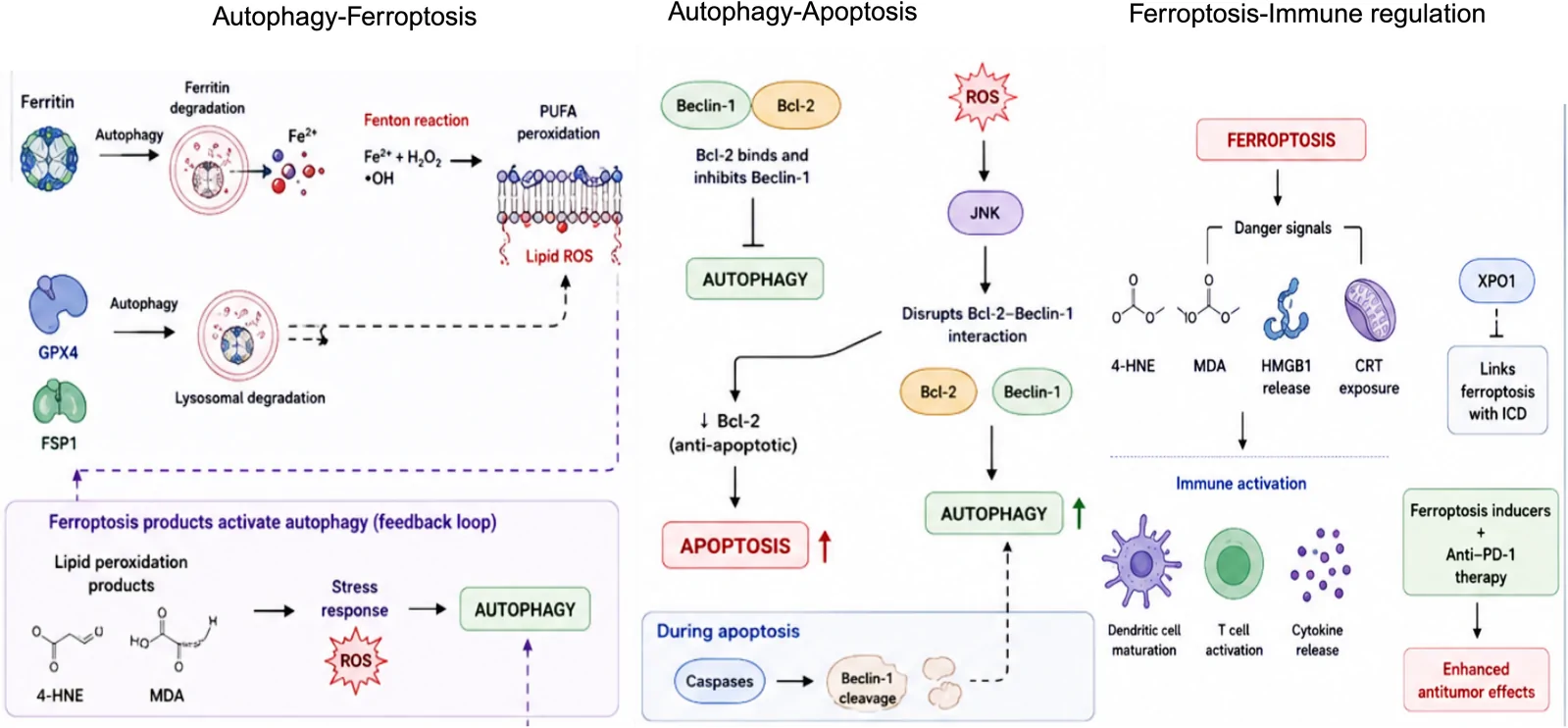
ROS-driven crosstalk among autophagy, ferroptosis, apoptosis, and ICD. Autophagy promotes ferroptosis through ferritin, GPX4, and FSP1 degradation, increasing iron-mediated lipid peroxidation. ROS-JNK signal links autophagy and apoptosis through disruption of the Bcl-2–Beclin-1 complex, while ferroptosis-derived signals activate antitumor immunity. Challenges, future direction, and conclusion.

Autophagy stimulates ferroptosis via degradation of ferritin, which releases labile iron to drive Fenton reaction and lipid peroxidation. Autophagic degradation of GPX4 and FSP1 promotes ferroptosis, while ferrostatin-1 and deferoxamine inhibit ferroptosis without affecting autophagy, stating that these are two distinct processes (**Figure 2**). 4-HNE and malondialdehyde—lipid peroxidation products of ferroptosis—activate autophagy as a stress response, making a feedback amplification loop (Hou et al., 2016). Bcl-2 links apoptosis and autophagy by binding and inhibiting Beclin-1. ROS-activated JNK disrupts the Bcl-2–Beclin-1 interaction and stimulates autophagy while downregulation Bcl-2’s anti-apoptotic activity (**Figure 2**). During apoptosis, caspase-mediated Beclin-1 cleavage further connects the two pathways (Palabiyik, 2025). Ferroptosis stimulates immune activation through 4-HNE, MDA, HMGB1 discharge, and CRT exposure. XPO1 links ferroptosis with ICD, and combining ferroptosis inducers with anti-PD-1 therapy has shown enhanced antitumor effects (**Figure 2**) (Conche et al., 2023). Thus, ROS integrates all four major cell death pathways, while excessive ROS can activate multiple cell death pathways to overcome therapy resistance, therefore demonstrating the critical function of ROS in cancer therapy. Pro-oxidant agents (anthracyclines, platinum-based drugs, arsenic trioxide, radiotherapy, and photodynamic therapies) utilize oxidative stress to induce tumor cell death. Nanoparticle-based platforms such as iron oxide nanoparticles, ROS-responsive nanocarriers, gold nanoparticles (Au@AgBiS_2_ core–shell structures), and mitochondria-targeted brequinar-loaded nanoparticles enhance the accuracy and efficacy of ROS delivery within the TME. Platycodin D2 inhibited breast cancer proliferation by inducing mtROS generation to trigger autophagy flux blockade and ferroptosis. Autophagy inhibition and ferroptosis mutually reinforced each other in an mtROS-dependent manner, resulting in enhanced anticancer effects (Li et al., 2025). Trifluoperazine inhibits oral cancer by triggering GPX4-mediated, autophagy-dependent ferroptosis through mitochondrial damage, lipid ROS accumulation, and suppression of the SLC7A11/GPX4 axis (Tsai et al., 2024). Disulfiram copper enhanced the antitumor efficacy of chemotherapy by inducing ROS-mediated ferroptosis and activating autophagy, in bladder tumor growth, with elevated lipid peroxidation and LC3B expression and decreased p62, ALDH1/2, GSH, and SOD (Sharma et al., 2025). Targeting antioxidant defense systems (NRF2, GPX4, and TXNRD) sensitizes cancer cells to oxidative damage and ferroptosis, signifying that combination strategies integrating ROS inducers with apoptosis, ferroptosis, autophagy, and immunotherapy can activate multiple cell death pathways and improve outcome.

As discussed earlier in the review ROS, autophagy and NRF2 work in coordination in carcinogenesis where ROS as secondary messengers modulate oncogenic signaling of lung cancer, breast cancer, melanoma, and CRC. Moderate ROS promote carcinogenesis and metastasis, while excessive ROS induce apoptosis or autophagic cell death. The duality of autophagy is seen in tumorigenesis, as a tumor suppressor during early stages, but promotes carcinogenesis in later stages of PDAC, breast, and thyroid cancers, by adapting to metabolic stress and chemotherapy (Shan et al., 2024). NRF2 is frequently hyperactivated through KEAP1 mutations or p62/SQSTM1-mediated signaling in lung cancer, liver cancer, and CRC, and persistent NRF2 activation reduces oxidative stress, inhibits apoptosis, and promotes resistance to therapy by NRF2 addiction. Low-dose ionizing radiation (LDIR) induces an adaptive radioresistant phenotype in lung cancer cells through coordinated activation of ROS, autophagy, ERK1/2, and the NRF2/HO-1 antioxidant pathway. Moderate ROS generated by LDIR stimulate autophagy and activate NRF2 and its downstream target HO-1. This antioxidant response enhances cellular redox homeostasis, promotes DNA repair, suppresses apoptosis, and increases resistance to subsequent high-dose radiation and chemotherapeutic stress (Chen et al., 2015). Inhibition of ROS, autophagy, ERK1/2, NRF2, or HO-1 abolishes the protective effect of LDIR, demonstrating that the ROS-autophagy-NRF2/HO-1 signaling causes LDIR-induced radio-resistance.

Despite significant achievements, there are several challenges that limit the clinical translation of ROS-based therapies. Intratumoral heterogeneity affects the substantial differences in redox status and antioxidant capacity among cancer cells and causes variable treatment responses and development of resistant populations. Targeting tumor cells in tissue is challenging due to the narrow therapeutic window. In addition, adaption of tumor cell to oxidative stress by activating antioxidant pathways reduces therapeutic efficacy. The extensive crosstalk among ferroptosis, autophagy, apoptosis, and ICD further complicates therapeutic interventions, as modulation of one pathway may influence others. The lack of reliable biomarkers (GPX4, ACSL4, LC3, Beclin-1, caspases, CRT, HMGB1, and ATP) and the need to optimize ICD-inducing therapies with immunotherapy remain major challenges for clinical translation. Thus, future studies should integrate patient-derived models, single-cell multiomics, AI-based drug discovery, and clinical evaluation of ROS-based immunotherapies while exploring the roles of metabolism, diet, and the microbiome in tumor redox regulation. Future research finds a scope in integrating redox nanomedicine, AI-assisted drug discovery, patient-derived models, and multiomics techniques to advance precision cancer therapy. Redox-responsive nanocarriers can improve targeted drug delivery while minimizing off-target toxicity. AI-driven platforms can accelerate the identification of novel redox targets and optimize drug combinations. Patient-derived organoids and xenograft models offer clinically relevant systems for evaluating treatment efficacy and predicting patient-specific responses. Furthermore, integrating genomics, transcriptomics, proteomics, metabolomics, and single-cell analyses will enable comprehensive characterization of tumor heterogeneity, facilitate biomarker discovery, and support the development of personalized therapeutic strategies, ultimately improving clinical outcomes.

In conclusion, ROS serve as central regulators that modulate an interconnected cell death network. Recent advances in elucidating the molecular mechanisms underlying redox signaling have identified multiple therapeutic targets, including the GPX4–SLC7A11–NRF2 axis, AMPK-mTOR signaling, mitochondrial apoptotic pathways, and ICD-associated DAMP signaling. The development of pro-oxidant therapies, antioxidant defense inhibitors, ferroptosis inducers, immunotherapy-based combinations, and ROS-responsive nanoplatforms advances the translational potential of redox-targeted interventions. Exploiting ROS-based multi-modal cell death may be an effective strategy to overcome tumor heterogeneity and therapeutic resistance and a foundation for next-generation cancer therapeutics.
